# Shifting the Paradigm: The Putative Mitochondrial Protein ABCB6 Resides in the Lysosomes of Cells and in the Plasma Membrane of Erythrocytes

**DOI:** 10.1371/journal.pone.0037378

**Published:** 2012-05-24

**Authors:** Katalin Kiss, Anna Brozik, Nora Kucsma, Alexandra Toth, Melinda Gera, Laurence Berry, Alice Vallentin, Henri Vial, Michel Vidal, Gergely Szakacs

**Affiliations:** 1 Institute of Enzymology, Research Centre for Natural Sciences, Hungarian Academy of Sciences, Budapest, Hungary; 2 Unité Mixte de Recherche 5235 (Dynamique des Interactions Membranaires Normales et Pathologiques), Centre National de la Recherche Scientifique, Université Montpellier II, Montpellier, France; Auburn University, United States of America

## Abstract

ABCB6, a member of the adenosine triphosphate–binding cassette (ABC) transporter family, has been proposed to be responsible for the mitochondrial uptake of porphyrins. Here we show that ABCB6 is a glycoprotein present in the membrane of mature erythrocytes and in exosomes released from reticulocytes during the final steps of erythroid maturation. Consistent with its presence in exosomes, endogenous ABCB6 is localized to the endo/lysosomal compartment, and is absent from the mitochondria of cells. Knock-down studies demonstrate that ABCB6 function is not required for de novo heme biosynthesis in differentiating K562 cells, excluding this ABC transporter as a key regulator of porphyrin synthesis. We confirm the mitochondrial localization of ABCB7, ABCB8 and ABCB10, suggesting that only three ABC transporters should be classified as mitochondrial proteins. Taken together, our results challenge the current paradigm linking the expression and function of ABCB6 to mitochondria.

## Introduction

ATP-binding cassette (ABC) transporters comprise a superfamily of membrane spanning multidomain proteins. ABC proteins are located in the membrane compartment of the cells, where they mediate translocation of various molecules across these barriers. The functional significance of ABC transporters is suggested by the evolutionary conservation of this protein superfamily. Based on sequence homology, 48 different ABC proteins (grouped into seven subfamilies ranging from A to G) have been defined in the human genome. Most human ABC proteins function as efflux pumps, translocating their substrates into the extracellular space or intracellular organelles. Human ABC transporters may be expressed in the plasma membranes of the cells, and also in intracellular membrane compartments including the endoplasmic reticulum (ER), lysosomes, peroxisomes and mitochondria. ABCB6 belongs to the B (MDR/TAP) sub-family of ATP-binding cassette transporters. The B subfamily consists of 11 members, with functions ranging from the translocation of phosphatidylcholine (ABCB4-MDR3) to secretion of bile acids (ABCB11) or the protection of cells against xenobiotics (ABCB1-MDR1/Pgp). The ABCB subfamily contains seven half transporters, of which four (ABCB6, ABCB7, ABCB8 and ABCB10) are believed to reside in the mitochondria. ABCB2 and ABCB3 (TAP1-2) act as heterodimers mediating the translocation of immunogenic peptides from the cytosol into the endoplasmic reticulum, ABCB9 has been localized to the lysosomes [1]–[2], and the cellular localization of ABCB5 remains to be clarified.

The exact physiological function of mitochondrial ABC transporters is not known. Presumably, they play a role in the transport of solutes across mitochondrial membranes (thus regulating the intramitochondrial milieu), or in the communication of signals transmitted from the mitochondria to the cell. The inner mitochondrial membrane ABC transporters most likely act as exporters: mutations of ABCB7 result in mitochondrial iron overload [3] (although the exact nature of the transported substrate remains elusive), and ABCB8 is also believed to be involved in mitochondrial iron export [4]. The high expression of ABCB10 in tissues related to hematopoiesis suggests a role in erythroid differentiation, with a function presumably contributing to the tight regulation of mitochondrial iron acquisition and heme synthesis [5].

The first report describing ABCB6 function was based on the investigation of a *Saccharomyces cerevisiae* mutant strain lacking the mitochondrial ABC transporter Atm1p [6]. Atm1p knockout cells accumulate high mitochondrial iron levels and are more sensitive to oxidative stress [7], [8], iron-starvation [9] and heavy metal toxicity [10]. Based on the analysis of this complex phenotype, Atm1p is believed to transport a yet unknown substrate that has an important role in the biogenesis of cytosolic/nuclear iron-sulfur proteins as well as in regulating the overall mitochondrial iron homeostasis [11], [12], [13]. In complementation studies using yeast strains lacking Atm1p, the human ABCB6 protein (formerly known as MTABC3) was shown to provide rescue from these phenotypic alterations. Based on this result, ABCB6 was suggested to be the human ortholog of Atm1p [6]. In 2006, Krishnamurthy and colleagues reported that ABCB6 catalyzes the mitochondrial uptake of coproporphyrin III (CPIII), an oxidized derivative of the heme synthesis intermediate, coproporphyrinogen III (CPgenIII), thereby serving as an important regulator of cellular porphyrin biosynthesis [14].

For several reasons, we consider both suggestions controversial. First, ABCB6 was assigned to the outer mitochondrial membrane with its nucleotide binding domains facing the cytoplasm. According the currently accepted model, this orientation implies an inward transport (i.e. mitochondrial import), which is difficult to reconcile with the export function of Atm1p that resides in the inner mitochondrial membrane. Second, the outer mitochondrial membrane is permeable to small molecules, in contrast to the inner membrane that represents an impermeable membrane barrier. Third, ABCB6 was shown to undergo glycosylation [15] and ER/Golgi transition [16]. In addition to its mitochondrial localization, ABCB6 was also detected in the plasma membrane [17], the Golgi apparatus [16], and in organelles of the vesicular system [18], [19], [20], [21].

While each ABC transporter may have a dedicated physiological function, their overall role seems to be tied to the “chemoimmunity” network providing resistance against xenobiotic stress (for more details see [22]–[23]). Thus, with the exception of a few notable examples (such as CFTR or SUR1/2), the pathophysiological conditions associated with ABC proteins are linked to the (lack of) transport of potentially toxic molecules. Accordingly, several ABC transporters act as “multidrug resistance” (MDR) proteins that provide resistance against chemotherapy in cancer [24]. Several reports suggest that the function of ABCB6 may be related to MDR pumps. Expression of ABCB6 correlates with increased resistance to drugs in the NCI60 cell panel [25] and various cell lines selected for resistance to arsenite, cisplatin, camptothecin were found to possess elevated levels of ABCB6 mRNA and protein [26]. Recently, overexpression of ABCB6 has been linked to clinical resistance in acute myeloid leukemia and to incomplete response to treatment in breast cancer [27]–[28]. However, as classical MDR pumps (including ABCB1/Pgp or ABCG2) function in the cell membrane to keep cytotoxic drugs below a cell-killing threshold, the MDR function of ABCB6 is also difficult to reconcile with its suggested mitochondrial localization and orientation.

To elucidate the role of ABCB6 in cellular physiology, it seems important to analyze and establish the exact subcellular localization of the native ABCB6 protein. Here we provide experimental evidence based on independent methods proving that the endogenous human ABCB6 is in fact an endolysosomal protein, which is also expressed in red blood cells. We confirm the mitochondrial localization of ABCB7, ABCB8 and ABCB10, suggesting that only these three ABC transporters should be classified as mitochondrial proteins. Taken together, our results challenge the current paradigm linking the expression and function of ABCB6 to mitochondria.

## Results

### ABCB6 is Expressed in the Endosomal/lysosomal Compartment and is Absent from Mitochondria

**Figure 1:**
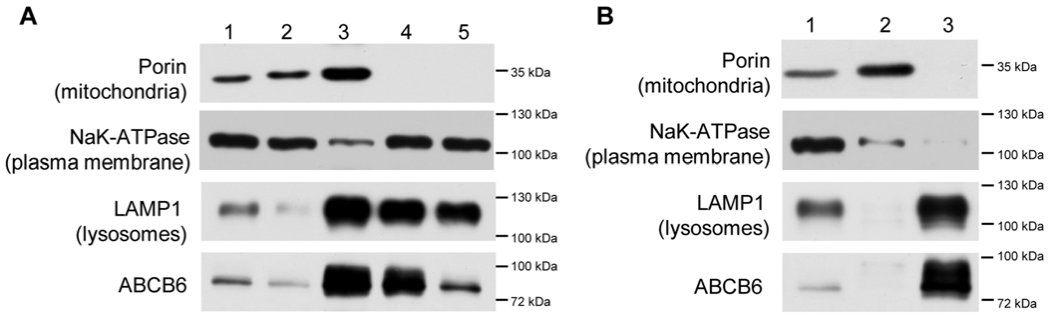
ABCB6 is not present in the purified mitochondrial fraction of K562 cells. Cellular membranes were separated by differential centrifugation (A); mitochondria were isolated using anti-TOM22 magnetic beads (B) as described in Materials and Methods. Following separation by SDS-PAGE, organelle membranes were transferred to a PVDF membrane, which was incubated in antibodies recognizing specific organelle markers and ABCB6. **A**. Cellular fractions corresponding to total cell lysate (lane 1); nuclei and intact cells (lane 2); mitochondrial pellet of the 8000×g centrifugation (lane 3); pellet of 12.000×g centrifugation (lane 4); pellet of 20.000×g centrifugation (lane 5). **B**. Isolation of mitochondria using TOM22 immunobeads. Total cell lysate (lane 1); fraction bound to the beads after repeated washing steps (lane 2); pellet of the flow through (lane 3).

K562, a Bcr-Abl+ erythroleukemia cell line is a widely used human model system for studying the molecular mechanisms implicated in hemoglobin production [29]. Based on experiments conducted with K562 cells, ABCB6 was suggested to mediate mitochondrial uptake of porphyrins [14]. In an effort to follow up on these studies, we used differential centrifugation and immunoaffinity purification to analyze membrane fractions of parental K562 cells; the endogenous ABCB6 protein was visualized using a highly specific monoclonal antibody [17] in the context of organelle markers. Although the mitochondria-containing fraction obtained by differential centrifugation was positive for ABCB6-labeling, mitochondrial localization of ABCB6 could not be ascertained due to the persistent contamination of the mitochondrial fraction with lysosomes (Figure 1A). To overcome difficulties associated with the overlapping physicochemical properties of organelles, we used an alternative approach that relies on superparamagnetic microbeads conjugated to anti-TOM22 antibody [30]. Membrane fractions isolated from K562 cell extracts by immunomagnetic separation were positive for porin but negative for LAMP-1, indicating mitochondria devoid of lysosomal contamination (Figure 1B). In that setting, the ABCB6 signal separated from the porin-positive fraction, and coincided with LAMP-1.

**Figure 2:**
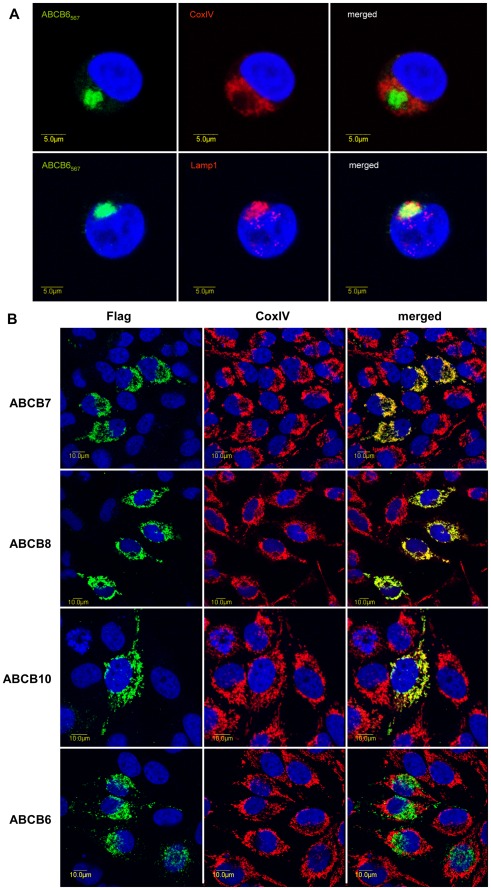
Determination of the subcellular localization of ABCB6 by double immunofluorescence labeling and laser-scanning confocal microscopy. A . Expression of the endogenous ABCB6 protein in K562 cells was visualized by the monoclonal ABCB6-567 antibody (green), lysosomes and mitochondria were labeled with LAMP1 (red, lower panel) and CoxIV antibodies (red, upper panel), respectively. ABCB6 colocalizes with LAMP1 (yellow on the overlay, r = 0.79), but not with CoxIV (r = 0.14). **B**. Flag-tagged ABC proteins were expressed following transient transfection of HeLa cells. The cDNA-derived ABC transporters were visualized with an anti-FLAG tag antibody (green, left panel); mitochondria were labeled with CoxIV (red, middle panel). Whereas the canonical mitochondrial ABC transporters are confined to the mitochondria (merged image, right panel, r = 0.87, r = 0.7, r = 0.84 for ABCB7, ABCB8 and ABCB10, respectively), ABCB6 does not show colocalisation with the mitochondrial marker (merged image, right panel, r = 0.13).

Immunocytochemical analysis of K562 cells by confocal microscopy confirmed the localization of the endogenous ABCB6 protein in the lysosomal compartment (labeled by LAMP1), and its absence in mitochondria (labeled by CoxIV) (Figure 2A). Further analysis of HeLa cells consistently showed that the endogenous ABCB6 protein colocalizes primarily with lysosomal markers, and is not expressed in the plasma membrane or mitochondria; HEK cells expressing endogenous ABCB6 gave identical results (Figure S1). To investigate whether the reported mitochondrial localization of ABCB6 may be explained by an artefact linked to overexpression systems used in those studies [14], [16], [17], [21], we transfected HeLa cells with a construct encoding a Flag-tagged ABCB6 variant. This experimental approach also resulted in an endolysosomal expression pattern that was clearly distinct from the mitochondrial expression of ABCB7, ABCB8 or ABCB10 (Figure 2B and Table S1). Interestingly, at very high expression levels, ABCB6 was also found in the plasma membrane, whereas under the same experimental conditions the canonical mitochondrial ABC transporters remained confined to the mitochondria (Figure S2).

### ABCB6 is Expressed as a Full Length Glycoprotein in RBCs

**Figure 3:**
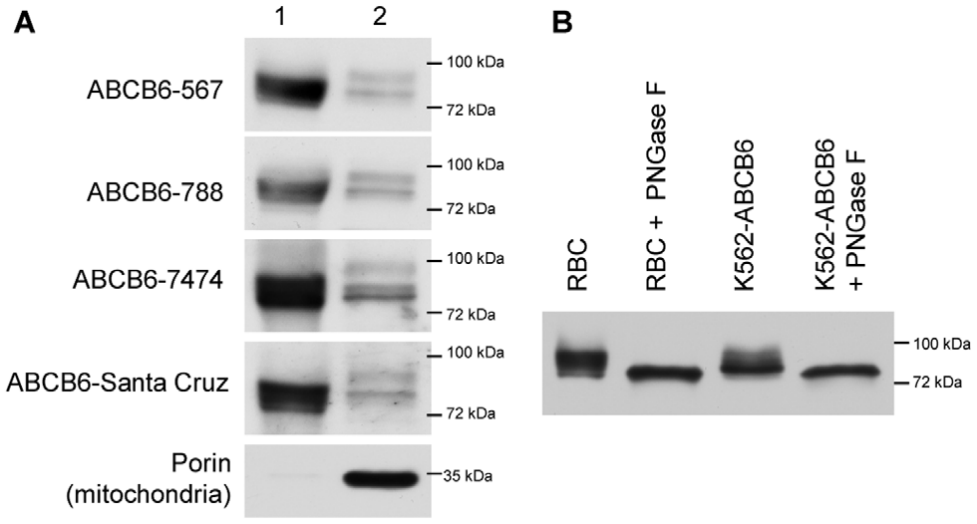
ABCB6 is expressed as a full length glycoprotein in mature erythrocytes. A . Red blood cell ghosts (60 ug total protein, lane 1) were solubilized and analyzed by sodium dodecyl sulfate-polyacrylamide gel electrophoresis (SDS-PAGE). After transfer to a PVDF membrane, Western blot analysis was performed using a panel of custom-made and commercially available anti-ABCB6 antibodies as well as anti-porin to exlude contamination by mitochondria. Membranes of K562 cells engineered to overexpress HA-tagged ABCB6 were loaded as a control (10 µg total protein, lane 2). **B**. Red blood cell ghosts and ABCB6 overexpressing K562 cell lysates were treated with PNGase F enzyme to remove N-glycans. 20 ug total protein was analyzed by SDS-PAGE, ABCB6 was visualized by the ABCB6-567 antibody.

ABCB6 is highly expressed in fetal liver, and ABCB6 protein levels were reported to increase coordinately with globin levels during the induced differentiation of proerythroblast cell lines [14]. To expand the scope of our analysis from cancer cell lines to models of higher physiological relevance, we analyzed terminally differentiated red blood cells. As shown in Figure 3A, Western blot analysis indicates that ABCB6 is expressed in mature erythrocytes; imnunofluorescence analysis of fresh human blood using two different anti-ABCB6 antibodies confirmed this result (Figure S3). Following treatment of erythrocyte ghosts with peptide N-glycosidase F (PNGaseF), a clear shift of the apparent molecular weight is observed, indicating that ABCB6 is expressed as an N-glycosylated protein in the plasma membrane of mature erythrocytes (Figure 3B). According to the conventional wisdom, mature red blood cells lack intracellular organelles. In fact, red blood cells are completely devoid of mitochondria, since mitochondria are removed in the final stages of erythroid differentiation [31]. Thus, the presence of a glycosylated ABCB6 in mature erythrocytes is inconsistent with the presumed mitochondrial localization of this protein in erythrocyte precursors.

### ABCB6 is Released in Exosomes from the Reticulocytes

As erythrocytes differentiate from reticulocytes, we assessed the presence and fate of ABCB6 in immature red blood cells. Mice were phlebotomized to increase their reticulocyte count in blood (up to 20%). Consistently, an increase in transferrin receptor (TfR) expression was displayed by circulating red blood cells without noticeable change in spectrin content (Figure S4A). Interestingly, RBCs from anemic mice presented much higher amounts of ABCB6 compared to those of healthy animals, suggesting a loss of the ABC transporter during reticulocyte maturation into erythrocyte. Such loss of specific proteins during reticulocyte maturation has been shown to occur through their association with exosomes. Exosomes correspond to intralumenal vesicles of internal compartments called multivesicular endosomes (MVE), secreted in the extracellular medium upon fusion of MVE with the plasma membrane [32]. Thus, reticulocytes from mice treated with phenylhydrazine were differentiated *in vitro* for 48 h and the secreted exosomes were collected from the maturation medium. The TfR and proteins classically taken as exosomal markers (Alix, Tsg101, flotillin) were found in the released vesicles. Remarkably, ABCB6 was also detected in the secreted exosomes and consistently the amount of cell-associated transporter decreased during in vitro maturation period, as in the case of the TfR (Figure S4B). Conversely, B-spectrin, which is not lost during reticulocyte maturation was not detected in exosomes [33]. Moreover, the absence of porin in the exosomal fraction rules out a contamination by mitochondria exocytosed during reticulocyte differentiation [31]. Accordingly, after reticulocyte fractionation, ABCB6 was recovered in plasma membrane, endosome and exosome subfractions (not shown).

**Figure 4:**
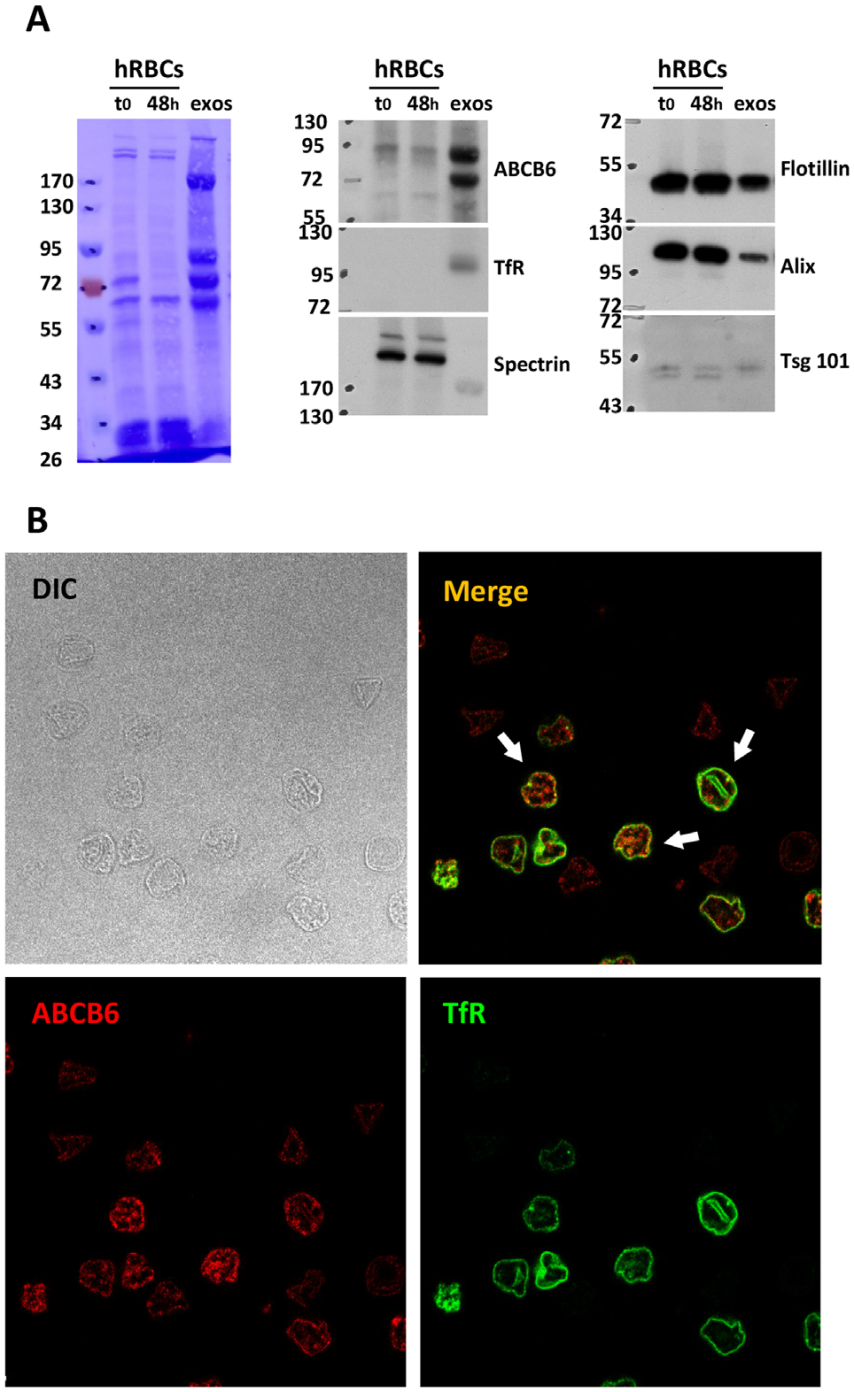
Human reticulocytes release ABCB6 in association with exosomes. A . 250 µL packed cells volume (PCV) of human RBCs from reticulocyte-enriched (4.5%) blood was cultured for 48 h and exosomes were collected from the medium as previously described in [42]. 0.5 µL PCV of RBCs before (t0) or after (48 h) maturation, as well as the completeness of exosomes were loaded on 10% SDS-PAGE for immunoblot analysis of the indicated proteins. The molecular mass (kDa) standards are indicated on the left. Note that the TfR is not detected in RBCs due to the low reticulocyte count but revealed in exosomes due to its concentration in the vesicles. **B**. Human RBCs from reticulocyte-enriched (10%) blood were adsorbed on cover slips pretreated with poly-L-lysine, fixed 20 min by 1% PFA and immunostained for ABCB6 (antibody Ab 7474 at; dilution 1/:50/Alexa Fluor 594; A21207) or and TfR (MoAb H68.4 at; dilution 1/:200/Alexa Fluor 488; A11029) after permeabilization, as described in the Materials and Methods. Coverslips were observed using a Leica confocal SPE and a Leica 63× ACS APO 1.33 objective. Note that none of the cells shown are positive to DAPI although contained in the mounting reagent. Arrows point to possible colocalization of ABCB6 and TfR in MVE.

Human RBCs from reticulocyte-enriched blood was similarly used for in vitro maturation (Figure 4A). Correspondingly, the vesicles containing the exosomal markers (and not spectrin) were enriched with TfR and ABCB6. Note that the exosomal enrichment of the two cargo proteins is especially noticeable due to the low reticulocyte count of human RBCs, accentuating the final TfR and ABCB6 concentration in exosomal membrane compared to RBCs plasma membrane. To confirm ABCB6 association with the vesicles, exosomes collected after reticulocyte maturation were fractionated by flotation on sucrose gradient and analyzed for the presence of TfR, Tsg101 and ABCB6 by Western blot (Figure S5). TfR and Tsg101 were mainly detected in fractions 5 to 8, corresponding to densities between 1.11 and 1.20 g/mL, in agreement with exosome characteristics [34]. Outstandingly, ABCB6 was distributed in the same fractions, attesting its exosome association. Immunofluorescence study was carried out on human RBCs and confirmed the higher expression of ABCB6 on TfR-positive reticulocytes (Figure 4B).

### ABCB6 is Coregulated with the Hemoglobin Content of Induced K562 Cells, but is Dispensable for the Erythroid Differentiation of K562 Cells

**Figure 5:**
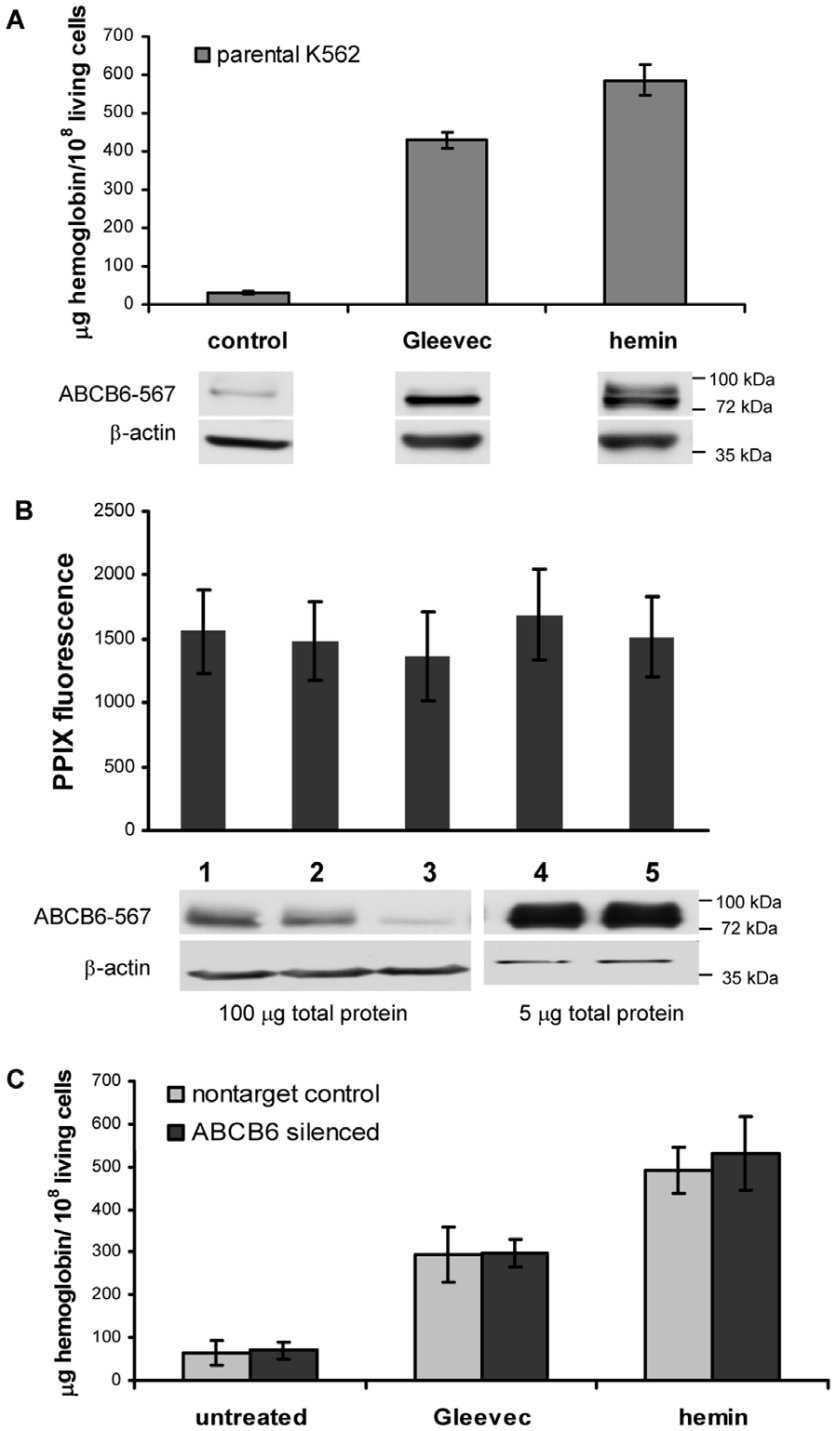
Porphyrin synthesis and ABCB6 expression in K562 cells. **A**. ABCB6 protein expression is upregulated in parental K562 cells upon chemically induced erythroid differentiation. K562 cells were cultured for 72 h with 150 nM Gleevec or 50 µM hemin, the number of viable cells was determined by trypan-blue staining. Hemoglobin levels were quantified by benzidine staining. Error bars represent SD of at least 5 experiments. ABCB6 expression is revealed by the ABCB6-567 monoclonal antibody (60 ug total protein, 72 hours post-induction). Actin is shown as loading control. **B**. Baseline PPIX-fluorescence of K562 cells is not influenced by ABCB6 levels. PPIX fluorescence was measured by flow cytometry as explained in the Methods section, ABCB6 expression of the cells was revealed by the anti human ABCB6-567 monoclonal antibody (actin is shown as loading control). Error bars represent SD of at least 3 experiments. Parental K562 cells (lane 1); K562 cells stably transfected with a non-target control shRNA (lane 2) or ABCB6 shRNA construct (lane 3); K562 cells stably overexpressing wild-type ABCB6 (lane 4) or a non-functional mutant variant of ABCB6 (ABCB6KM, lane 5). **C**. Downregulation of ABCB6 does not impede induced heme synthesis of K562 cells. K562 cells stably transfected with an shRNA construct targeting ABCB6 or a scrambled control were cultured for 72 h with 150 nM imatinib or 50 µM hemin, hemoglobin levels were quantified by benzidine staining. Error bars represent SD of at least 5 experiments.

K562 cells can be differentiated along the erythroid lineage by a variety of chemical compounds including hemin, and various antitumor agents [29]. We investigated differentiating K562 cells treated with the targeted Bcr-Abl tyrosine kinase inhibitor, Gleevec (imanitib mesylate) [35] or hemin. Since imatinib is toxic to K562 cells, experiments were conducted at conditions that result in maximal hemoglobin production without inducing apoptosis. At 72 hours after the induction with 150 nM imatinib or 50 µM hemin, parental K562 cells contained elevated quantities of hemoglobin as evidenced by the visible change in the color of the cellular pellets (Figure S6) and an increase of benzidine staining (Figure 5A). At 72 hours post induction, ABCB6 protein levels were significantly increased – confirming that ABCB6 expression is upregulated during erythroid maturation and increased hemoglobin production [14].

It was hypothesized that the elevated expression of ABCB6 would promote mitochondrial porphyrin uptake and activate de novo porphyrin biosynthesis, which can be readily followed experimentally by measuring the protoporphyrin IX (PPIX) fluorescence of the cells [14]. To verify this proposition, we studied the effect of ABCB6 overexpression or shRNA-mediated downregulation on the baseline PPIX-levels of stably transfected K562 cells. Despite the high-yield overexpression of the wild type ABCB6 protein or the effective downregulation of the endogenous ABCB6 levels by inducible expression of short hairpin RNAs, baseline PPIX-fluorescence of the different K562 cell lines cells did not change significantly (Figure 5B). Finally, we characterized the effect of ABCB6 silencing on the de novo heme synthesis of induced K562 cells. Downregulation of ABCB6 protein levels did not change the ability of Gleevec or hemin to enhance hemoglobin production (Figure 5C), suggesting that ABCB6 is dispensable for the induced de novo heme biosynthesis required for the chemically induced erythroid differentiation of K562 cells.

## Discussion

Of the ∼1500 proteins identified in the erythrocytes, proteomic studies have confirmed the presence of several ABC transporters, suggesting that the mature erythrocyte membrane is a major repository of ABC proteins [36]. Here we show that ABCB6, a protein currently assigned to mitochondria in the major protein databases [37], is abundantly expressed in the erythrocyte membrane. Our findings expand red blood cell proteomic data that include ABCB6 fragments [38]–[40]. We show that ABCB6 is expressed in its full length, glycosylated form, suggesting that the plasma membrane localization in red blood cell does not rely on erythrocyte-specific posttranslational modifications. The presence of ABCB6 in red blood cells may seem logical in view of the increased expression of ABCB6 in erythroid differentiation models (Figure 5A and [14]). However, since mature erythrocytes do not contain intracellular membrane compartments, plasma membrane localization of ABCB6 is inconsistent with the presumed mitochondrial localization of this protein in erythrocyte precursors. Intracellular organelles such as mitochondria and endosomes are lost during the final stage of differentiation through autophagy and secretion [41]. In fact, the red blood cell ghosts are negative for mitochondrial markers such as porin (Figure 3). We show that ABCB6 is at least partly evacuated through exosome secretion. Since exosomal cargo proteins must originate from the plasma membrane-endosomal continuum, this result provides further arguments against the presumed mitochondrial localization of ABCB6. By filtering components to be secreted *vs.* components to be recycled to the plasma membrane, exosome biogenesis also contributes to RBC plasma membrane remodeling. Similarly to the lysosomal protein LAMP2 [42], ABCB6 could be partly redistributed to the plasma membrane of mature erythrocytes during reticulocyte maturation by fusion of lysosomes with the plasma membrane [43]. The functional relevance of such “neolocalized” cell surface proteins in RBCs awaits further investigation.

At present, it is uncertain whether erythrocytic ABCB6 is actually a bioactive molecule or a vestigial heritage from an erythroid precursor. Similarly to ABCB7 and ABCB10, expression of ABCB6 was shown to increase upon DMSO-induced differentiation of mouse erythroleukemia (MEL) cells [44], suggesting that these proteins may all share an important role in the regulation of the heme synthetic pathway. While our data do not exclude the possibility that ABCB6 may play a role in heme metabolism under certain conditions that remain to be defined, the results presented in this paper suggest that it does not mediate direct mitochondrial uptake of porphyrins.

First, our morphological data show that ABCB6 is not identified in purified mitochondria. Second, we show that the endogenous and cDNA-derived human ABCB6 (NM_005689) is targeted to the endolysosomal compartment, unlike ABCB7, ABCB8 or ABCB10, which were found in the mitochondria. Third, ABCB6 is glycosylated, and to date only one mammalian glycoprotein has been described in mitochondria, although this number could be underestimated [45]. Finally, we show that differential expression of ABCB6 does not influence baseline or induced porphyrin levels in K562 cells.

It is well known that overexpression takes a toll on the processing of proteins; tags may also derail intracellular targeting. Whereas most localization data in the literature are based on the overexpression of tagged ABCB6 protein forms [6], [14], [16], [17], [21], we characterized the intracellular distribution of the endogenous protein, in three human cell lines of different origin, using complementary methods. We used a panel of custom-made and commercially available antibodies. The specificity of the antibodies was confirmed by immunoblotting and confocal microscopy studies of cell lines expressing tagged variants of ABCB6 (Figure S2, [17]). The major advantage of using antibodies to visualize the subcellular localization of proteins is that genetic constructs are not needed and that the possible artifactual effects of protein fusions are avoided. However, while fusion proteins may enable monitoring of dynamic changes, antibodies detect only a snapshot of the dynamic process of protein distribution. Immuncytochemical labeling of the ABCB6 protein with the currently available antibodies requires fixed and permeabilized cells, limiting the study to dynamically fixed end points. Under these conditions, the distribution of the native ABCB6 primarily shows colocalisation with lysosomes, and less so with the Golgi/ER markers (Table S2), presumably because of the short half-life of ABCB6 in the latter organelles (glycosylation proves that ABCB6 transits through the ER-Golgi system [15], [17]). Conversely, lack of mitochondrial localization, especially in the context of the observed expression pattern of ABCB7, ABCB8 and ABCB10 in the same experimental system indicates that ABCB6 does not reside in mitochondria.

K562 is a Philadelphia-positive erythroleukemia cell line that has been widely used to study induced hemoglobin production, even if this model does not fully recapitulate the complex physiology of terminal erythropoiesis [29]. In K562 cells, targeted inhibition of the oncogenic kinase initiates the entire differentiation program leading to enhanced *de novo* heme biosynthesis and ultimately to increased cellular hemoglobin levels [35]. In our hands, treatment with hemin or the Bcr-Abl kinase inhibitor Gleevec induced hemoglobin production and differentiation of K562 cells toward the erythroid lineage, which was accompanied by a significant increase of ABCB6 protein levels. However, overproduction or downregulation of ABCB6 did not result in a shift of baseline PPIX levels, and stable silencing by inducible shRNA did not prevent differentiation-induced hemoglobin upregulation of K562 cells. Notably, in a similar experimental setup, silencing of ABCB10 interfered with the ability of K562 cells to undergo induced differentiation [46].

Careful analysis of the literature provides additional support to our conclusions. A recent publication suggests that ABCB6 can be silenced without a consequent decrease of cellular PPIX levels [47]. Interestingly, abcb6-KO mice are viable and appear to be hematologically normal, suggesting that ABCB6 is dispensable (Jill Paterson, Gergely Szakacs, Michael M. Gottesman, unpublished data). While this manuscript was in preparation, ABCB6 was identified as the genetic basis of the Lan blood group antigen expressed on red blood cells. Of note, Lan(-) (ABCB6(−/−)) individuals do not suffer any clinical consequences, suggesting that ABCB6 is dispensable for erythropoiesis in humans [48]. Heterologous expression of rat Abcb6 in a human adenocarcinoma cell line resulted in a subcellular localization confined to the endo/lysosomal compartment [21]. ABCB6 was also found to be expressed in the plasma membrane [17]. While we don’t see evidence of the plasma membrane expression of ABCB6 in the cell lines analyzed in this study, we note that at high expression levels, cDNA-derived ABCB6 appears in the plasma membrane of HeLa cells. The continuum of the endolysosomal and plasma membrane compartments, as well as the abundance of ABCB6 in red cells adds further relevance of this finding. Data assembled by recent proteomic efforts contain reference to lysosomal localization [20]. Lysosomes are organelles of eukaryotic cells that are critically involved in the degradation of macromolecules mainly delivered by endocytosis and autophagy. The lysosomal membrane facilitates interaction and fusion with other compartments and harbors transport proteins catalyzing the export of catabolites, thereby allowing their recycling [49]. Given the ATP-dependence of ABC transporters, the ATP-binding domain of ABCB6 is expected to face the cytoplasm, predicting ABCB6 to be a lysosomal importer. In addition to Atm1p, ABCB6 is also homologous to the heavy metal tolerance factor 1 (HMT1) of *Caenorhabditis elegans* and *Schizosaccharomyces pombe*. HMT1 is a half transporter that confers tolerance to heavy metal toxicity in *Schizosaccharomyces pombe* [50] and tolerance to cadmium in *Caenorhabditis elegans* [51]. Notably, HMT1 is a vacuolar protein, which protects cells against metal toxicity by promoting the sequestration of metal adducts into the vacuolar interior. Interestingly, a possible role of ABCB6 in metal tolerance has been suggested in the literature. Direct correlation between arsenic resistance and ABCB6 expression was observed in various human and mouse cell lines [17], [52]. Knockdown of ABCB6 expression sensitized HepG2 and Hep3B cells to arsenite toxicity, and stable overexpression of ABCB6 conferred a strong survival advantage towards arsenite-induced oxidative stress [53]. These results are consistent with the prediction that ABCB6, as a functional orthologue of HMT1, pumps transition metal complexes into the interior of acidic vesicles.

Further work is needed to elucidate the functional relevance of ABCB6 in the pathophysiology of erythroid cells. It is possible that ABCB6 offers protection of the erythrocyte by excreting heme degradation products. In addition to the erythrocytic membrane, the snapshots of our morphological studies locate ABCB6 in the endo-lysosomal compartment of cells. This compartment includes different membrane organelles that interact together through specific targeting mechanisms and fusion processes in order to degrade, sort and recycle material of different origin. Interestingly, ABCB6 and TfR are expressed in the same endosomal compartment in HeLa cells (not shown), which is consistent with a colocalisation in an intracellular compartment of the erythroid precursors such as the multivesicular endosome, bringing about exosome production. In the complex dynamics of a living cell, endosomes may transiently contact mitochondria as part of the organelle-mediated delivery of iron observed in reticulocytes [54], [55], [56]. Could this transient interaction account for the reported mitochondrial localization and presumed function of ABCB6 in cell lines? The findings presented in this paper do not support this notion. While we do not exclude the contribution of ABCB6 to porphyrin metabolism, our results clearly show that ABCB6 is unlikely to be directly responsible for the mitochondrial uptake of porphyrins. We also show that ABCB6 function is not required for de novo heme biosynthesis in differentiating K562 cells, excluding this ABC transporter as a key regulator of porphyrin synthesis. At the same time we confirm the mitochondrial localization of ABCB7, ABCB8 and ABCB10, suggesting that only three ABC transporters should be classified as mitochondrial proteins. Taken together, our results challenge the current paradigm linking the expression and function of ABCB6 to mitochondria. The identification of the subcellular localization of ABCB6 should pave the way for studies aimed at the elucidation of its true physiological function.

## Materials and Methods

### Ethics Statement

Animal protocols were reviewed and approved by the Institutional Animal Care and Use Committee of the University Montpellier II. Reticulocyte-enriched blood (always >4%) was obtained from patients with pathological states, such as hemolytic anemia, during routine follow-up visits to the “Hôpital St Eloi” (Montpellier, France), as approved by the Montpellier University Hospital’s ethics committee. Control blood was obtained from healthy volunteers, as approved by the Montpellier University Hospital’s ethics committee. All subjects gave their written informed consent to participate in the study, which was approved by the Montpellier University Hospital’s ethics committee.

### DNA Constructs

cDNA encoding wild-type human ABCB6 (NM_005689) bearing an HA-tag at its C-terminus [17] was mutated by mutagenic PCR to generate a variant harboring mutation of a universally conserved lysine residue in the Walker A sequence (K629M). The HA-tagged ABCB6 and the non-functional ABCB6-variant (ABCB6HA and ABCB6HA-KM, respectively) were cloned into pBabe retroviral vectors (AddGene plasmid 1764). An untagged form of both the wild-type and the non-functional mutant variant of ABCB6 (K629M, ABCB6-KM) were cloned into pSEW lentiviral vectors. The pLKO.1-puro3×LacO inducible shRNA vectors targeting human ABCB6 or expressing a nonsense hairpin were purchased from Sigma. For transient transfection, a C-terminally Flag-tagged ABCB6 construct was created by PCR mutagenesis and subcloning into a 3×FLAG-CMV-14 vector (Sigma-Aldrich). pCMV-ABCB7, pCMV-ABCB8 and pCMV-ABCB10, containing C-terminally Flag-tagged constructs were kind gifts from Jill Paterson.

### Cell Lines, Culture Conditions

The K562 cell line (American Type Culture Collection) was kindly provided by Bela Papp, Inserm, Paris. Cells were grown in RPMI medium without nucleosides, supplemented with 10% (v/v) fetal bovine serum (Invitrogen Cat.No. 10106-169) and with 2 mM glutamine, 100 units/mL penicillin, and 100 µg/mL streptomycin (Invitrogen-Gibco, Carlsbad, CA, USA) at 37°C in humidified air/CO2 (19∶1) atmosphere. ABCB6-HA and its non-functional KM mutant variant (ABCB6-HA KM) were expressed in K562 cells using retroviral transduction. Briefly, the Phoenix-eco packaging cell line was transfected by using the ExGene transfection system (Fermentas). The cell-free viral supernatant was collected at 48 hours after transfection, and was immediately used to transduce PG13 cells. The Phoenix-eco cell line [57] was a gift from G. Nolan (Department of Pharmacology, Stanford University, Stanford, CA); PG13 retrovirus producing cells were obtained from ATCC (Manassas, VA). For the generation of ABCB6 knock-down cell lines, lentiviral particles were produced by following the manufacturer’s instructions. To induce the expression of the shRNA constructs, IPTG (1 mM) was added to the cells for 48 hours before additional treatments. Untagged variants of ABCB6 were stably expressed in K562 cells by lentiviral transduction as described above, and transiently expressed in HeLa cells (Sigma) following the instructions of the FuGENE HD Transfection kit (Promega). Since we found that Mycoplasma infection significantly influences the baseline fluorescence of cells (thus interfering with measurements of protoporphyrin IX (PPIX) levels), cell lines were regularly screened with the Mycoplasm Detection Kit (Lonza), and the assays were carried out in mycoplasma-negative cells.

### Antibodies

The following primary antibodies were used in Western blotting experiments: ß-actin (A1978, Sigma-Aldrich); ABCB6-567, ABCB6-788 and ABCB6-7474 [17], ABCB6 (Sc98685, Santa Cruz); porin (A31855, Invitrogen); LAMP1 (611043, BD Biosciences); NaK-ATPase (BML-SA247-0100, Biomarker); CoxIV (A21348, Invitrogen); Lac-I (AP09404PU-S, Acris antibodies, Germany); BXP21; TfR (H68.4, Invitrogen); Flotillin-1 (610820, BD Biosciences); Tsg 101 (4A10, Abcam); Alix (R. Sadoul, INSERM U836, Grenoble) and B-spectrin (N. Mohandas, NY Blood Center). HRP-conjugated secondary antibodies were purchased from Jackson Immuno Research Laboratories. The following primary antibodies were used for confocal microscopy: anti-Flag (F3165, Sigma); panCadherin (Ab16505, Abcam); giantin (Ab24586, Abcam); Lamp1 (L1418, Sigma); LAMP1 (611043, BD Bioscience); calnexin and CoxIV (Cellular Localization IF Antibody Sampler Kit, Cell Signaling, #4753). Fluorescently labeled secondary antibodies (goat anti-mouse IgGs conjugated with Alexa Fluor 647 A21235, Alexa Fluor 594 A11005 or Alexa Fluor 488 A10667; goat anti-rabbit IgGs conjugated with Alexa Fluor 647 A21244, Alexa Fluor 594 A11012 or Alexa Fluor 488 A11008) were purchased from Invitrogen.

### Differentiation of K562 Cells, Measurement of Baseline PPIX Fluorescence

K562 cells (2×10^5^/mL) were treated with various drugs for 3 days. Cells were grown beyond the logarithmic growth phase (*i.e.* over 10^6^cells/mL); diluted to 2×10^5^ cells/mL and treated with Gleevec immediately. For hemin treatments, the inducer was added to the diluted cultures after a four hour-long lag period; hemin was solubilised as described [58]. Special attention was given not to disturb air/CO2 (19∶1) atmosphere throughout the differentiation process. Viability was determined by trypan blue exclusion. Hemoglobin content of K562 cells was determined by the benzidine technique of Luftig [59] et al. with minor modifications: cells were lysed in a lysis buffer (50 mM Tris pH = 7,4; 250 mM NaCl; 2% Nonidet-P 40) at a density of 5×10^7^/ml. The hemoglobin content of clear supernatants was determined as described previously [60]. PPIX fluorescence was measured using an Attune acoustic focusing cytometer (Applied Biosystems, Life Technologies Corp. USA) equipped with a 405 nm (violet) laser and a 640 nm longpass filter.

### Confocal Microscopy

K562 cells were gently washed with Dulbecco’s modified phosphate-buffered saline (DPBS), fixed with 4% paraformaldehyde (PFA) in DPBS for 10 minutes at room temperature, dried on histopathological slides in drops, washed with DPBS, and permeabilized in methanol for 90 seconds at room temperature. The samples were blocked for 1 h at room temperature in DPBS containing 2 mg/mL BSA, 1% fish gelatin, 0.1% Triton-X 100 and 5% goat serum. The cells were then incubated for 1 h at room temperature with the primary antibody diluted in blocking buffer. After washing with DPBS, the cells were incubated for 1 hour at room temperature with the respective Alexa Fluor-conjugated secondary antibody diluted at 1∶250 in blocking buffer. Where indicated, DAPI was diluted in DPBS and added to the cells after the incubation with the secondary antibody, for 10 minutes at room temperature. Wheat Germ Agglutinin was added to living cells before the immunostaining procedure and incubated for 10 min at 37°C. To label cellular organelles, the following dyes were used: MitoTracker Red CMXos fixable dye (Invitrogen, M7512), LysoTracker Red DND-99 (Invitrogen, L7528), DAPI (4′,6-diamidino-2-phenylindole, dihydrochloride, D1306) and Alexa Fluor 633 conjugate of WGA (W21404). Samples were studied with an Olympus IX-81/FV500 laser scanning confocal microscope, using an Olympus PLAPO 60× (1.4 NA) oil immersion objective (Olympus Europa GmbH). Dual channel colocalization analysis was performed by the ImageJ software with the Colocalization Threshold and Colocalization Test plugins. Dual channel colocalization analysis was performed by the ImageJ software with the Colocalization Threshold and Colocalization Test plugins. Averaged Manders’ and Pearson’s coefficients are reported for each subcellular marker from 5 random pictures manders [61]–[62].

### Isolation of Erythrocytes and Reticulocytes

Human blood was freshly drawn into heparin from healthy volunteers. Cells were spun at 10000 rpm at 4°C for 10 min. The plasma and the buffy coat were removed by suction, and the red blood cells were washed three times with PBS. Reticulocyte-enriched blood (always >4%) was obtained from patients with pathological states, such as hemolytic anemia, during routine follow-up visits to the “Hôpital St Eloi” (Montpellier, France), as approved by the relevant institutional review boards. After centrifugation, the plasma and the buffy coat were removed, and the red blood cells were washed twice with Ringer solution. Anemia was induced in swiss-OF1 mice *via* two injections of an aqueous solution of phenylhydrazine hydrochloride (PHZ) (6 mg/mL) at the dose 60 mg/kg of on days 1 and 2. Mice were then sacrificed on day 6 by cardiac puncture under anaesthesia. Alternatively, reticulocytosis was induced by phlebotomy. Blood (0.5 ml) was removed from retroorbital vein on days 0 and 2. On day 4, animals were sacrificed and blood was collected by cardiac puncture. After removing the buffy coat, red blood cells were washed twice with Ringer solution, and used for fractionation or *in vitro* maturation. For control experiments, erythrocytes were obtained from the blood of untreated mice.

### Isolation of Organelle Membranes

Cellular fractions were isolated by differential centrifugation. Cells were washed with ice cold PBS and were resuspended in buffer A [10 mM NaCl, 1.5 mM MgCl2, 10 mM Tris (pH 7.4)] containing protease inhibitors (8 µg/mL aprotinine, 10 µg/mL leupeptine, 50 µg/mL PMSF) and 2 mM DTT, swollen on ice, and disrupted with a Dounce homogenizer (150 strokes). Buffer B [525 mM mannitol, 175 mM sucrose, 12.5 mM Tris (pH 7.4), and 2.5 mM EDTA] was added in a ratio of 4∶10 homogenate/buffer B (cell lysate: Figure 1A, lane 1). The nuclei and unbroken cells were removed by a low speed centrifugation (1300×g) for 10 min at 4°C as described in [63] (Figure 1A, lane 2). This step was repeated until no pellet was visible. The clear supernatant was centrifuged at medium speed (8000×g, 20 min) to get a fraction enriched in mitochondria (Figure 1A, lane 3) and the supernatant was further centrifuged at 12000×g for 30 min. The resulting pellet was free of mitochondria (Figure 1A, lane 4). The supernatant was further centrifuged at 20000×g for 45 min (Figure 1A, lane 5). The pellet of each separation step was dissolved in Laemmli buffer (0,05 M Tris-PO4 (pH: 6.8), 2% SDS, 2% B-merchaptoethanol, 0,002 M EDTA (pH 6.8), 20% glycerol, 0,02% bromphenol blue in ultra pure water) and sonicated for 12 sec. Equal amounts (protein) were subjected to SDS PAGE analysis.

Mitochondria were isolated using Miltenyi’s Mitochondria Isolation Kit (cat.no: 130-094-532), with minor modifications of the protocol to increase the purity of the mitochondria. Briefly, 2×10^7^ cells were washed with ice cold PBS and resuspended in 1 mL Lysis Buffer supplemented with 8 µg/ml aprotinine, 10 µg/mL leupeptine and 50 µg/mL PMSF (Sigma). Cells were broken in a Dounce homogenizer (150 strokes) and a 29G needle (10 strokes) (Figure 1B, lane 1). Nuclei and unbroken cells were removed with a low speed centrifugation step (5 min, 250×g, 4°C). 800 µL cell lysate was mixed with 6.3 mL of Separation Buffer and 30 µL anti-TOM22 MicroBeads was added to label mitochondria. The mixture was incubated for 60 min at 4°C in a tube rotator. The labeled cell lysate was transferred into a 4.5 mL polystyrene tube, which was placed into a magnetic separator column (EasySep, StemCell Technologies) for 10 minutes at 4°C. Following magnetic separation the supernatant was removed and the beads were washed with isolation buffer (3 mL). After 3 cycles of separation and washing, the solution containing labeled mitochondria was centrifuged at 12000×g 10 min, 4°C, the pellet was resuspended in Laemmli buffer (Figure 1B, lane 2). The supernatants from the washing steps were pooled and centrifuged at 10000×g for 10 min; the resulting supernatant was centrifuged at 18000×g for 30 min at 4°C, the pellet was subjected to SDS PAGE (Figure 1B, lane 3).

### Reticulocyte Subcellular Fractionation

Mouse red blood cells were lysed by freezing/thawing as described previously [64]. The lysed cells were pelleted at 1500×g for 5 min and the supernatant was centrifuged (30 000 rpm for 15 min in a Beckman TLA110 rotor) to pellet mitochondria and cell debris. An ultracentrifugation (70000 rpm for 2 h in a Beckman TLA110 rotor) finally allowed separating endosomes (pellet) from cytosol (supernatant). Endosomes were resuspended in PBS. Erythrocyte and reticulocyte plasma membranes were prepared according to Steck and Kant [65] with red blood cells from healthy and anemic mice, respectively. Animal protocols were reviewed and approved by the Institutional Animal Care and Use Committee of the University Montpellier II.

### Exosome Isolation

Red blood cells were cultured for 48 h at 37°C in RPMI 1640 supplemented with 5 mM glutamine, 5 mM adenosine, 10 mM inosine, 3% FCS, depleted for endogenous exosomes by overnight ultracentrifugation (100 000×g), 50 U/ml penicillin and 50 µg/ml streptomycin. After pelleting the cells, the culture supernatant was centrifuged (20 000×g for 20 min) to remove cellular debris. Exosomes were separated from the supernatant by ultracentrifugation (100 000×g for 2 h) and resuspended in sucrose 250 mM, Hepes 5 mM pH 7.4 or directly in Laemmli buffer, depending on the experiments. Exosomes collected from the 48 h maturation medium of human reticulocyte-enriched red blood cells (RBCs) were layered on top of a linear sucrose gradient (0.5–2.5 M sucrose) in a Beckman SW55 tube. Gradients were centrifuged at equilibrium for 16 h at 39 000 rpm, after which 350 µL fractions were collected from the top of the tube. Fraction densities were determined by refractometry. Proteins in collected fractions were precipitated by TCA, and samples were separated by SDS-PAGE using 10% polyacrylamide gels and the proteins were electrophoretically transferred to PVDF membrane (Immobilon-P; Millipore, Bedford, MA). Membranes were blocked for 1 hour in TBST (10 mM Tris–HCl, pH 8.0, 150 mM NaCl, 0.05% Tween 20) containing 5% skim milk, followed by 1 h incubation with the indicated primary antibodies. Blots were washed and incubated for 1 h at room temperature with the secondary antibody (horseradish-peroxidase (HRP)-conjugated). Immunoreactive bands were visualized by the enhanced chemiluminescence method (ECL, Amersham Bioscience) according to standard procedures. The membranes were stripped by overnight incubation with 0.2 M glycine, 0.1% SDS, 1% Tween 20, pH 2.2 and re-probed after washing with TBST and blocking with 5% skim milk.

## Supporting Information

Figure S1 **Determination of the subcellular localization of endogenous ABCB6 by double immunofluorescence labeling and laser-scanning confocal microscopy.** A. HeLa cells probed by the ABCB6-567 antibody to detect the endogenous ABCB6 protein (green) in the context of intracellular markers (red): CoxIV (mitochondria), panCadherin (plasma membrane), giantin (Golgi), calnexin (ER) and LysoTracker (lysosomes). ABCB6 colocalizes essentially with the lysosomal marker. B. HEK cells probed by the ABCB6-567 antibody to detect the endogenous ABCB6 protein (green) in the context of intracellular markers (red): WGA (plasma membrane), CoxIV (mitochondria) and Lamp1 (lysosomes). ABCB6 colocalizes essentially with the lysosomal marker.
(TIF)

Figure S2 **Flag-tagged ABCB6 expressed in HeLa cells.** Following transient transfection of HeLa cells with Flag-tagged ABCB6, cells were probed with anti-tag and ABCB6-567 antibodies. Proteins recognized by the antitag antibody are also labeled with ABCB6-567, suggesting that the monoconal antibody ABCB6-567 specifically recognizes ABCB6. Note that at very high expression levels, ABCB6 can also be found in the plasma membrane in addition to its main intracellular localization (bottom panel).
(TIF)

Figure S3 **Imnunofluorescence analysis of fresh human blood using two different anti-ABCB6 antibodies: 74740 (A) and Santa Cruz (B), both revealed with an Alexa 594-coupled secondary antibody.** A control with the secondary only is shown (C). DIC: differential interference contrast. Fresh human RBC (<48 h after sampling) group O+ were obtained from the French Blood center. The cells were washed with PBS and fixed in PBS with 4% of paraformaldehyde (EMS sciences) 4 hours at room temperature (RT). Cells were washed, treated with 0.1 M glycine in PBS for 15 minutes at (RT), and then permeabilized with 0.1% Triton X-100 in PBS for 10 minutes at RT. The cells were washed once and resuspended in 3% fetal calf serum (FCS). Antibodies were diluted in Washing Solution (PBS 1% FCS). The cells were incubated with the primary antibodies for 1 h at RT. After 3 washes, the cells were incubated with the secondary antibodies (anti-rabbit alexa 594, Molecular Probes) for 1 h and washed 3 times. A thin film was made on a glass slide and mounted with a coverslip and one drop of vectashield (Vector). Observations were made using a Zeiss Axioimager equipped with an apotome, with a 63× apochromat objective and Differential interference contrast. Luminosity and contrasts were adjusted using the Axiovision software.
(TIF)

Figure S4 **ABCB6 expression and fate during reticulocyte maturation. A**. Proteins from 0.5 µL packed cell volume (PCV) of RBCs from healthy or phlebotomized mice were separated on 10% SDS-PAGE, transferred on PVDF membrane and analyzed by Western blot for the presence of the indicated proteins after membrane staining/destaining using Coomassie blue. The molecular mass (kDa) standards are indicated on the right. **B**. 200 µL PCV of RBCs from PHZ-treated mouse was cultured for 48 h and exosomes were collected from the medium as described in the Materials and Methods. 0.5 µL PCV of RBCs before (t0) or after (48 h) maturation, and the completeness of exosomes were loaded on 10% SDS-PAGE for immunoblot analysis of the indicated proteins. Note that the 55 kDa band detected by the anti-ABCB6 (567) in RBCs and exosomes was frequently found (4/8 mice) in PHZ-treated mice and could correspond to a degradation product or to a shorter form reported by Paterson et al [17]. It has to be noted that additional bands may represent nonspecific reactions between the antibody generated against human ABCB6 and various murine proteins.
(TIF)

Figure S5 **Sucrose gradient analysis of hRBC exosomes.** Exosomes were obtained after in vitro maturation (48 h) of hRBCs (4% reticulocytes) by differential centrifugation, and layered on top of a linear sucrose gradient (0.5–2.5 M sucrose) in a Beckman SW55 tube. Gradients were centrifuged at equilibrium for 16 h at 39 000 rpm, after which 350 µL fractions were collected from the top of the tube. Fractions were collected and analyzed by Western blot for the indicated proteins. Densities (g/mL) were obtained for each fraction by refractometry and are indicated under each lane. Note that a significant amount of protein was detected on top of the gel by the anti-ABCB6 (657), which indicates that part of the transporter could form aggregates during TCA precipitation.
(TIF)

Figure S6 **Pelleted K562 cells.** 72 hours after the induction with 150 nM imatinib or 50 µM hemin, K562 cells contained elevated quantities of hemoglobin as evidenced by the visible change in the color of the cellular pellets.
(TIF)

Table S1 **Statistics of colocalization analysis between the mitochondrial marker CoxIV, ABCB6 and the mitochondrial ABC proteins.** Flag-tagged ABC proteins were expressed following transient transfection of Hela cells. The cDNA-derived ABC transporters were visualized with an anti-FLAG tag antibody, mitochondria were labeled with CoxIV. Dual channel colocalization analysis was performed by the ImageJ software with the Colocalization Threshold and Colocalization Test plugins. Averaged Pearson’s coefficients were calculated for each subcellular marker from 5 random pictures.
(DOC)

Table S2 **Colocalization of endogenous ABCB6 and different subcellular markers in HeLa cells.** LysoTracker was used as lysosomal, pancadherin as plasma membrane, calnexin as ER, giantin as Golgi and CoxIV as mitochondrial marker, as described in Methods section.
(DOC)
