# Supplementary figures and images for: Shifting the Paradigm: The Putative Mitochondrial Protein ABCB6 Resides in the Lysosomes of Cells and in the Plasma Membrane of Erythrocytes
Source: PLoS One. 2012 May 24;7(5):e37378. doi: 10.1371/journal.pone.0037378 (PMC3360040; doi:10.1371/journal.pone.0037378)

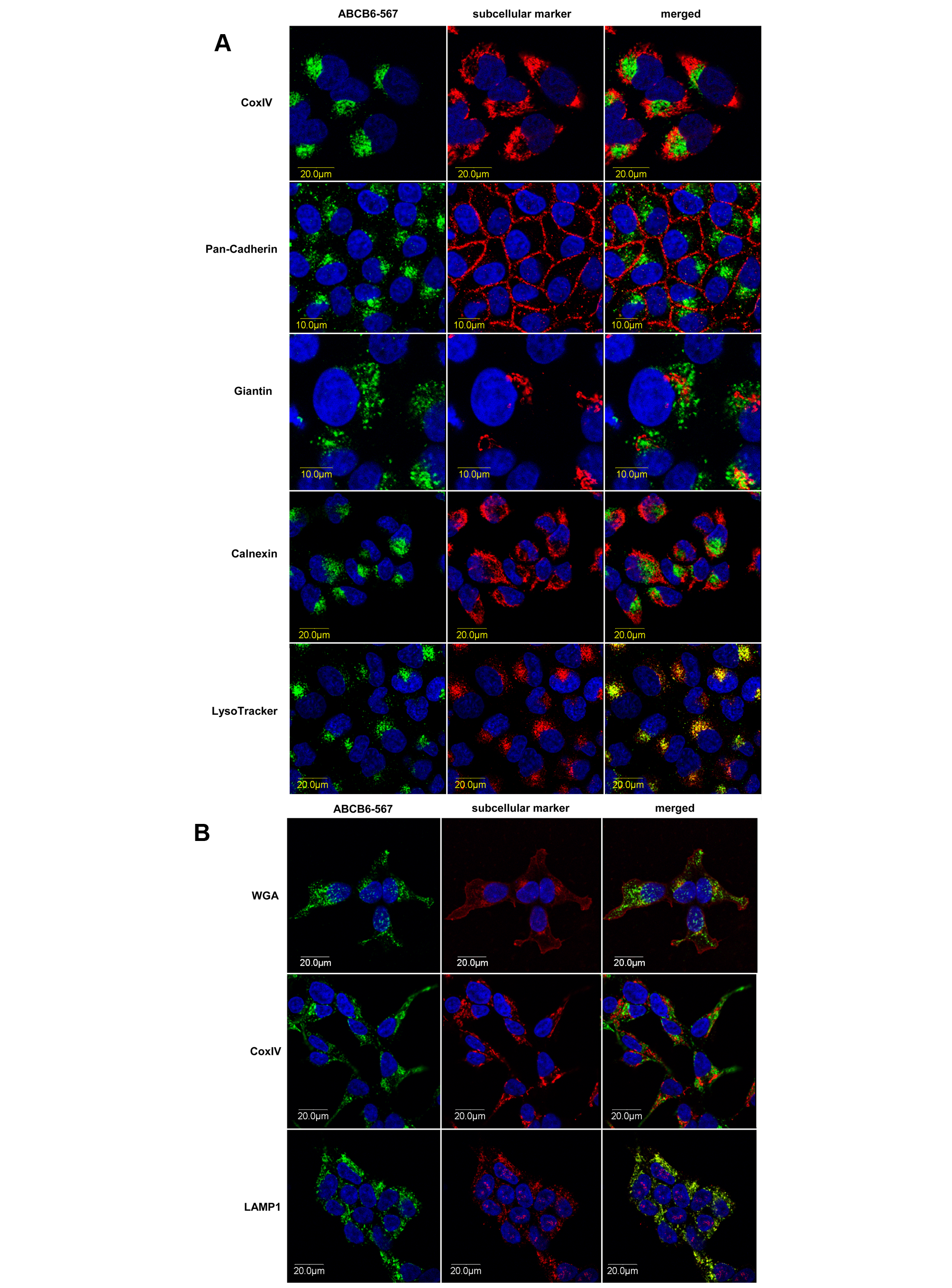

Supplement: Figure S1 — Determination of the subcellular localization of endogenous ABCB6 by double immunofluorescence labeling and laser-scanning confocal microscopy. A. HeLa cells probed by the ABCB6-567 antibody to detect the endogenous ABCB6 protein (green) in the context of intracellular markers (red): CoxIV (mitochondria), panCadherin (plasma membrane), giantin (Golgi), calnexin (ER) and LysoTracker (lysosomes). ABCB6 colocalizes essentially with the lysosomal marker. B. HEK cells probed by the ABCB6-567 antibody to detect the endogenous ABCB6 protein (green) in the context of intracellular markers (red): WGA (plasma membrane), CoxIV (mitochondria) and Lamp1 (lysosomes). ABCB6 colocalizes essentially with the lysosomal marker. (TIF) [file pone.0037378.s001.tif]

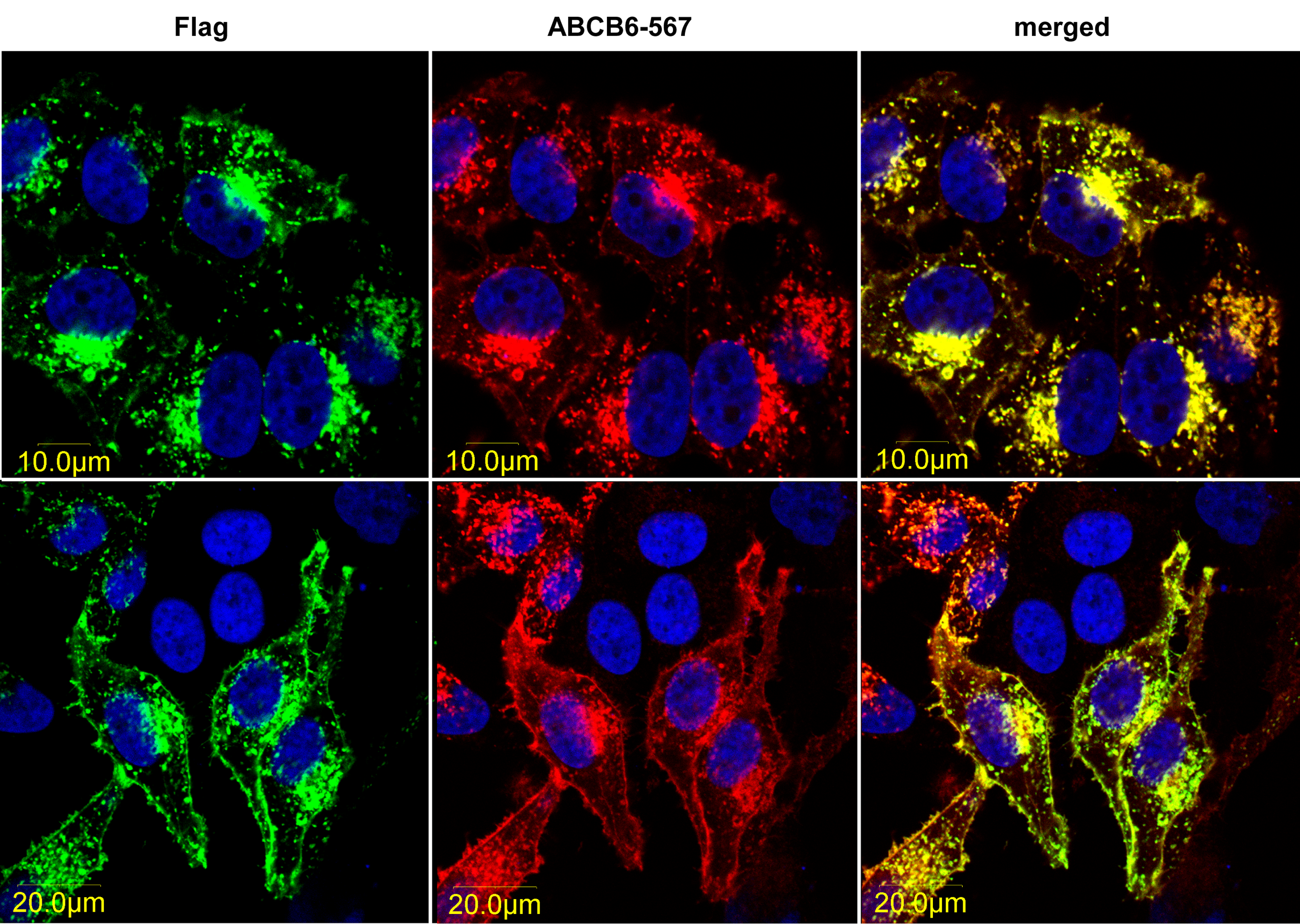

Supplement: Figure S2 — Flag-tagged ABCB6 expressed in HeLa cells. Following transient transfection of HeLa cells with Flag-tagged ABCB6, cells were probed with anti-tag and ABCB6-567 antibodies. Proteins recognized by the antitag antibody are also labeled with ABCB6-567, suggesting that the monoconal antibody ABCB6-567 specifically recognizes ABCB6. Note that at very high expression levels, ABCB6 can also be found in the plasma membrane in addition to its main intracellular localization (bottom panel). (TIF) [file pone.0037378.s002.tif]

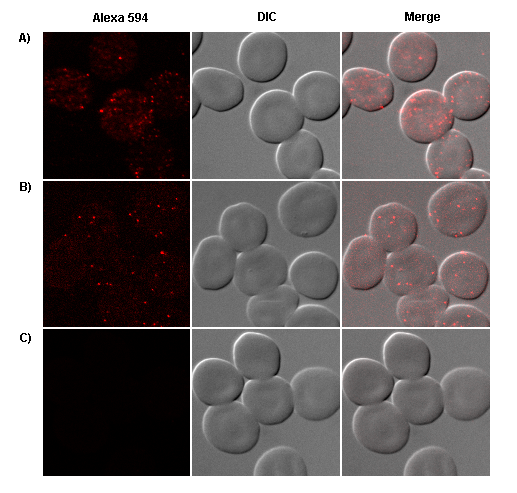

Supplement: Figure S3 — Imnunofluorescence analysis of fresh human blood using two different anti-ABCB6 antibodies: 74740 (A) and Santa Cruz (B), both revealed with an Alexa 594-coupled secondary antibody. A control with the secondary only is shown (C). DIC: differential interference contrast. Fresh human RBC (<48 h after sampling) group O+ were obtained from the French Blood center. The cells were washed with PBS and fixed in PBS with 4% of paraformaldehyde (EMS sciences) 4 hours at room temperature (RT). Cells were washed, treated with 0.1 M glycine in PBS for 15 minutes at (RT), and then permeabilized with 0.1% Triton X-100 in PBS for 10 minutes at RT. The cells were washed once and resuspended in 3% fetal calf serum (FCS). Antibodies were diluted in Washing Solution (PBS 1% FCS). The cells were incubated with the primary antibodies for 1 h at RT. After 3 washes, the cells were incubated with the secondary antibodies (anti-rabbit alexa 594, Molecular Probes) for 1 h and washed 3 times. A thin film was made on a glass slide and mounted with a coverslip and one drop of vectashield (Vector). Observations were made using a Zeiss Axioimager equipped with an apotome, with a 63× apochromat objective and Differential interference contrast. Luminosity and contrasts were adjusted using the Axiovision software. (TIF) [file pone.0037378.s003.tif]

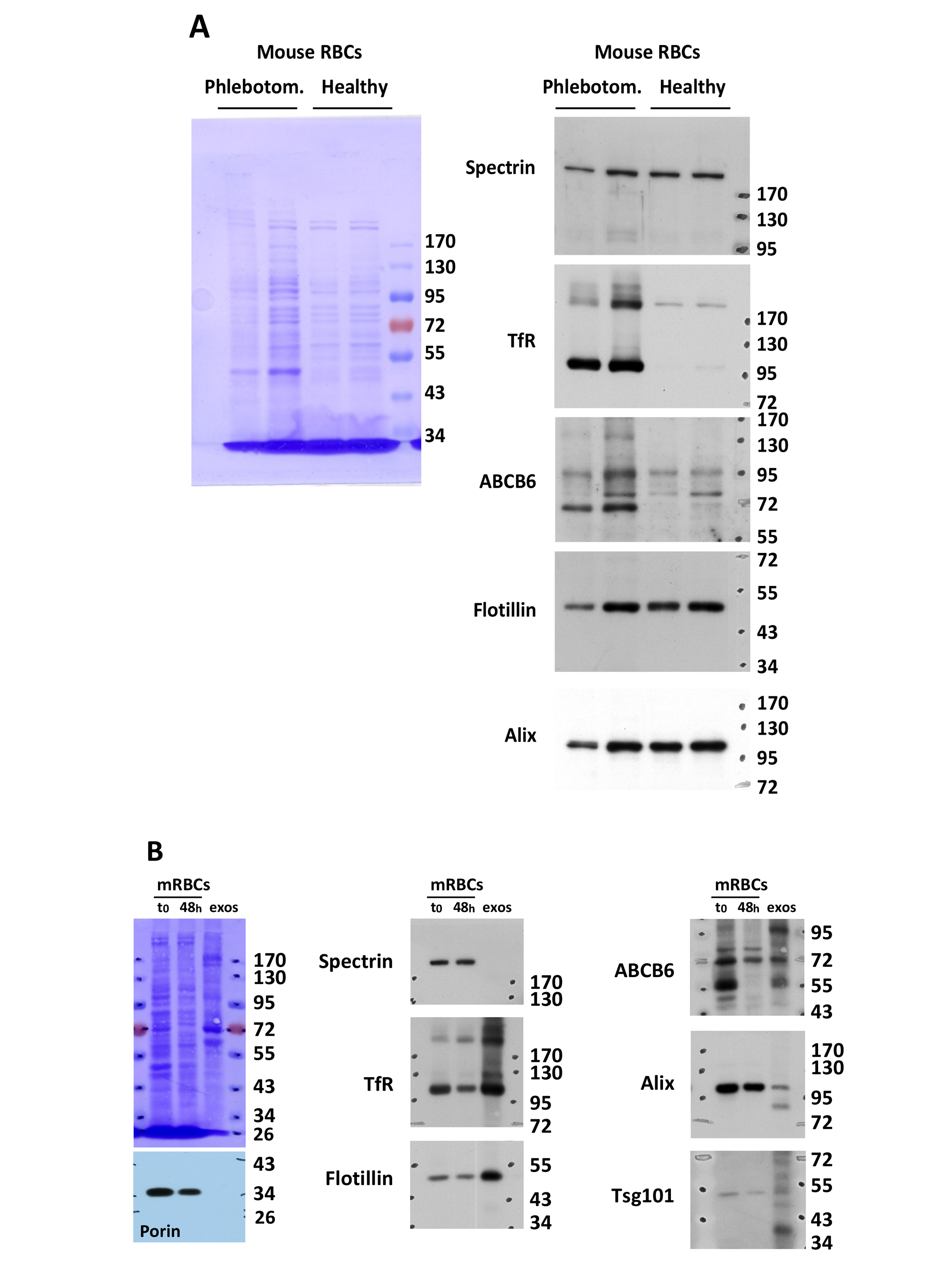

Supplement: Figure S4 — ABCB6 expression and fate during reticulocyte maturation. A. Proteins from 0.5 µL packed cell volume (PCV) of RBCs from healthy or phlebotomized mice were separated on 10% SDS-PAGE, transferred on PVDF membrane and analyzed by Western blot for the presence of the indicated proteins after membrane staining/destaining using Coomassie blue. The molecular mass (kDa) standards are indicated on the right. B. 200 µL PCV of RBCs from PHZ-treated mouse was cultured for 48 h and exosomes were collected from the medium as described in the Materials and Methods. 0.5 µL PCV of RBCs before (t0) or after (48 h) maturation, and the completeness of exosomes were loaded on 10% SDS-PAGE for immunoblot analysis of the indicated proteins. Note that the 55 kDa band detected by the anti-ABCB6 (567) in RBCs and exosomes was frequently found (4/8 mice) in PHZ-treated mice and could correspond to a degradation product or to a shorter form reported by Paterson et al [17]. It has to be noted that additional bands may represent nonspecific reactions between the antibody generated against human ABCB6 and various murine proteins. (TIF) [file pone.0037378.s004.tif]

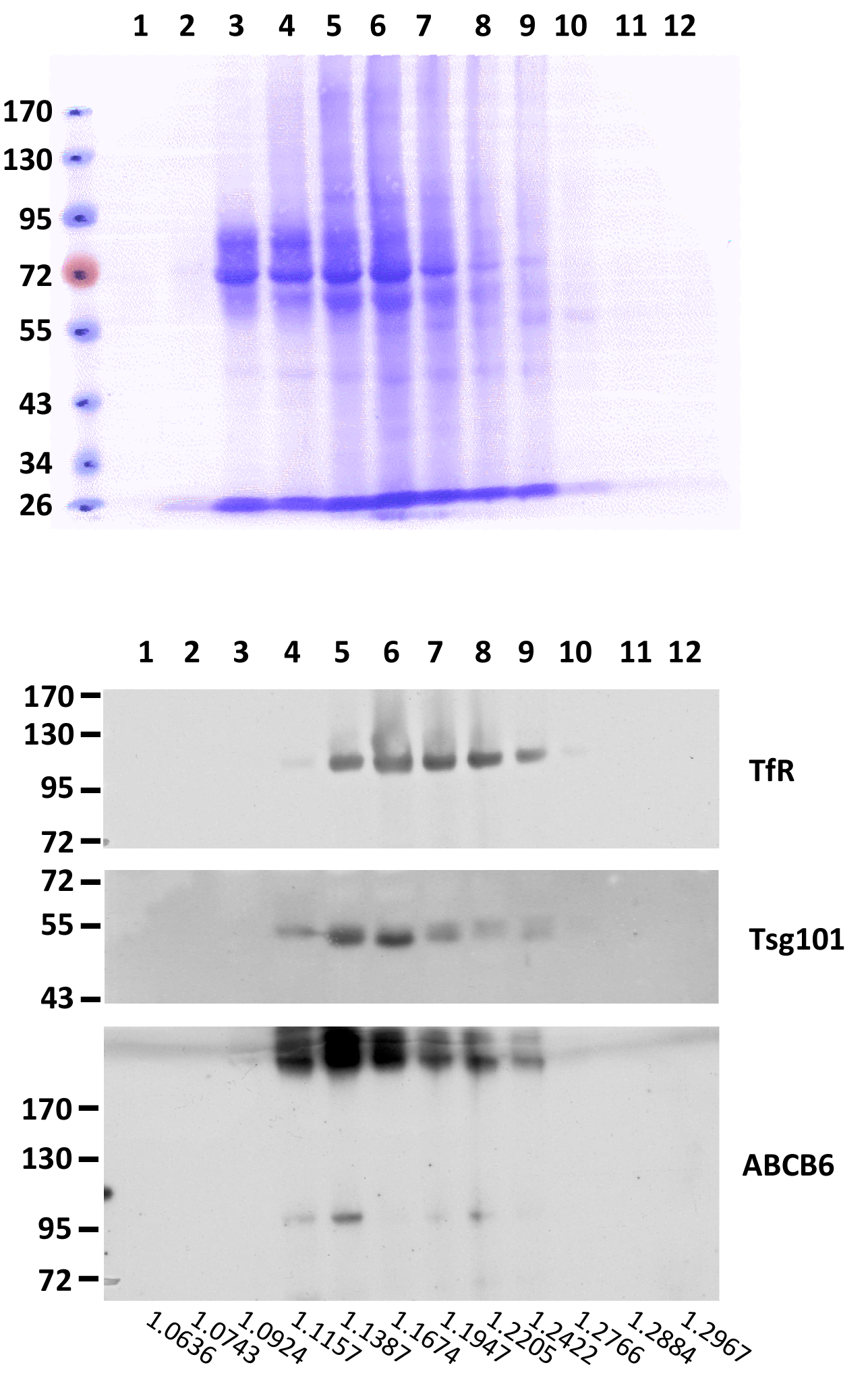

Supplement: Figure S5 — Sucrose gradient analysis of hRBC exosomes. Exosomes were obtained after in vitro maturation (48 h) of hRBCs (4% reticulocytes) by differential centrifugation, and layered on top of a linear sucrose gradient (0.5–2.5 M sucrose) in a Beckman SW55 tube. Gradients were centrifuged at equilibrium for 16 h at 39 000 rpm, after which 350 µL fractions were collected from the top of the tube. Fractions were collected and analyzed by Western blot for the indicated proteins. Densities (g/mL) were obtained for each fraction by refractometry and are indicated under each lane. Note that a significant amount of protein was detected on top of the gel by the anti-ABCB6 (657), which indicates that part of the transporter could form aggregates during TCA precipitation. (TIF) [file pone.0037378.s005.tif]

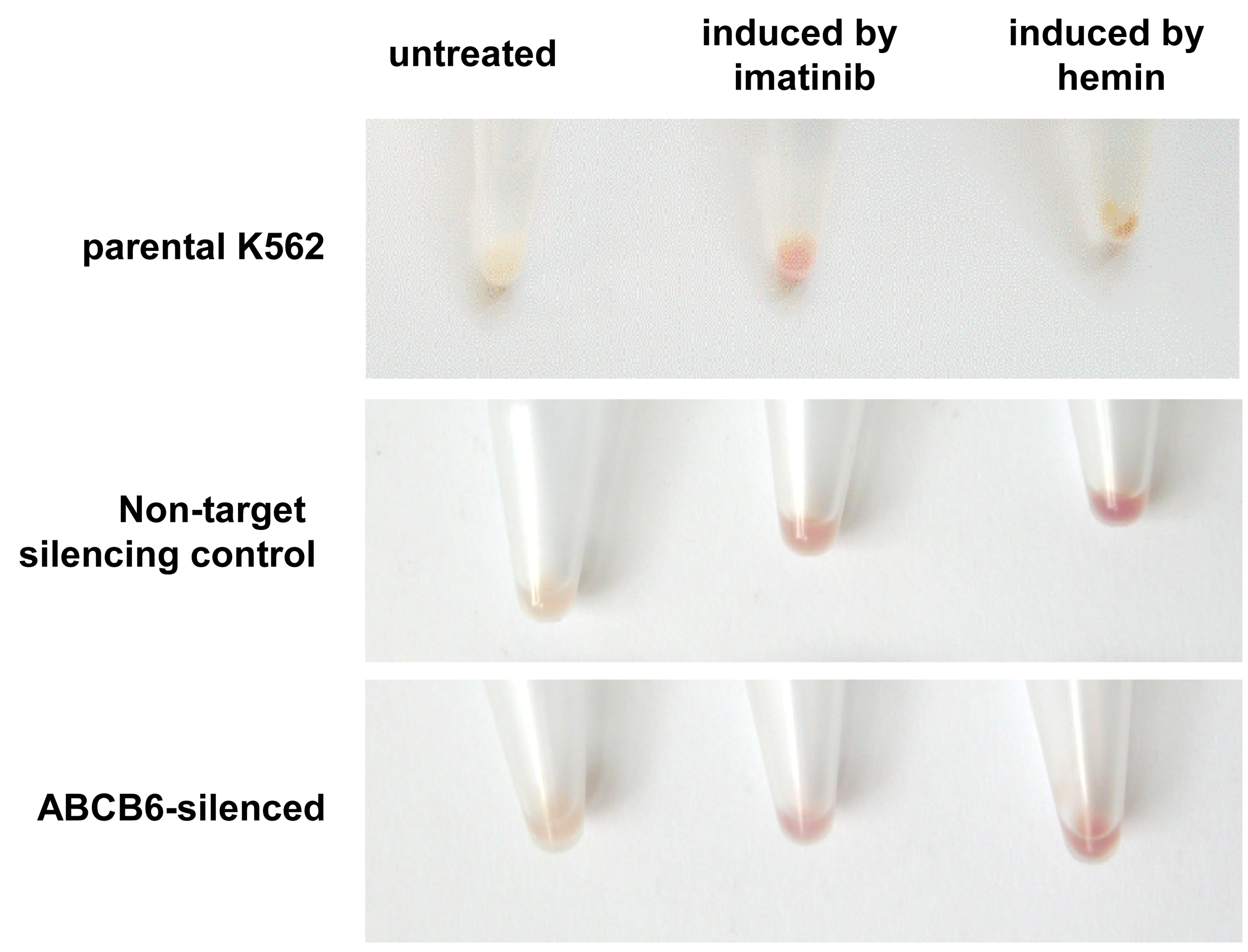

Supplement: Figure S6 — Pelleted K562 cells. 72 hours after the induction with 150 nM imatinib or 50 µM hemin, K562 cells contained elevated quantities of hemoglobin as evidenced by the visible change in the color of the cellular pellets. (TIF) [file pone.0037378.s006.tif]
