# Supplementary material for: Shifting the Paradigm: The Putative Mitochondrial Protein ABCB6 Resides in the Lysosomes of Cells and in the Plasma Membrane of Erythrocytes
Source: PLoS One. 2012 May 24;7(5):e37378. doi: 10.1371/journal.pone.0037378 (PMC3360040; doi:10.1371/journal.pone.0037378)
Supplement: Table S1 — Statistics of colocalization analysis between the mitochondrial marker CoxIV, ABCB6 and the mitochondrial ABC proteins. Flag-tagged ABC proteins were expressed following transient transfection of Hela cells. The cDNA-derived ABC transporters were visualized with an anti-FLAG tag antibody, mitochondria were labeled with CoxIV. Dual channel colocalization analysis was performed by the ImageJ software with the Colocalization Threshold and Colocalization Test plugins. Averaged Pearson’s coefficients were calculated for each subcellular marker from 5 random pictures. (DOC) [file pone.0037378.s007.doc]

**Supplementary Table 1: Statistics of colocalization analysis between the mitochondrial marker CoxIV, ABCB6 and the mitochondrial ABC proteins.**

|  | ABCB6 | | ABCB7 | | ABCB8 | | ABCB10 | |
| --- | --- | --- | --- | --- | --- | --- | --- | --- |
|  | average | SD | average | SD | average | SD | average | SD |
| Pearson’s coefficient | 0,130 | 0,100 | 0,870 | 0,045 | 0,696 | 0,127 | 0,836 | 0,044 |
| Overlapping coefficient | 0,176 | 0,087 | 0,908 | 0,038 | 0,796 | 0,081 | 0,895 | 0,023 |

Flag-tagged ABC proteins were expressed following transient transfection of Hela cells. The cDNA-derived ABC transporters were visualized with an anti-FLAG tag antibody, mitochondria were labeled with CoxIV. Dual channel colocalization analysis was performed by the ImageJ software with the Colocalization Threshold and Colocalization Test plugins. Averaged Pearson’s coefficients were calculated for each subcellular marker from 5 random pictures.
