# Supplementary material for: Shifting the Paradigm: The Putative Mitochondrial Protein ABCB6 Resides in the Lysosomes of Cells and in the Plasma Membrane of Erythrocytes
Source: PLoS One. 2012 May 24;7(5):e37378. doi: 10.1371/journal.pone.0037378 (PMC3360040; doi:10.1371/journal.pone.0037378)
Supplement: Table S2 — Colocalization of endogenous ABCB6 and different subcellular markers in HeLa cells. LysoTracker was used as lysosomal, pancadherin as plasma membrane, calnexin as ER, giantin as Golgi and CoxIV as mitochondrial marker, as described in Methods section. (DOC) [file pone.0037378.s008.doc]

**Supporting Table S2.** Colocalization of endogenous ABCB6 and different subcellular markers in HeLa cells.

|  | Pearson’s coefficient | | Overlapping coefficient | |
| --- | --- | --- | --- | --- |
|  | average | SD | average | SD |
| lysosome | 0.702 | 0.077 | 0.756 | 0.091 |
| plasma membrane | 0.111 | 0.030 | 0.249 | 0.015 |
| ER | 0.411 | 0.054 | 0.532 | 0.043 |
| Golgi | 0.494 | 0.016 | 0.552 | 0.018 |
| mitochondria | 0.210 | 0.123 | 0.181 | 0.049 |

LysoTracker was used as lysosomal, pancadherin as plasma membrane, calnexin as ER, giantin as Golgi and CoxIV as mitochondrial marker, as described in Methods section.
